# Lexical Knowledge Boosts Statistically-Driven Speech Segmentation

**DOI:** 10.1037/xlm0000567

**Published:** 2018-06-28

**Authors:** Shekeila D. Palmer, James Hutson, Laurence White, Sven L. Mattys

**Affiliations:** 1 Department of Psychology, University of York; 2 School of Psychology, Plymouth University; 3 Department of Psychology, University of York

**Keywords:** speech segmentation, statistical learning, lexical knowledge

## Abstract

The hypothesis that known words can serve as anchors for discovering new words in connected speech has computational and empirical support. However, evidence for how the bootstrapping effect of known words interacts with other mechanisms of lexical acquisition, such as statistical learning, is incomplete. In 3 experiments, we investigated the consequences of introducing a known word in an artificial language with no segmentation cues other than cross-syllable transitional probabilities. We started with an artificial language containing 4 trisyllabic novel words and observed standard above-chance performance in a subsequent recognition memory task. We then replaced 1 of the 4 novel words with a real word (*tomorrow*) and noted improved segmentation of the other 3 novel words. This improvement was maintained when the real word was a different length to the novel words (*philosophy*), ruling out an explanation based on metrical expectation. The improvement was also maintained when the word was added to the 4 original novel words rather than replacing 1 of them. Together, these results show that known words in an otherwise meaningless stream serve as anchors for discovering new words. In interpreting the results, we contrast a mechanism where the lexical boost is merely the consequence of attending to the edges of known words, with a mechanism where known words enhance sensitivity to transitional probabilities more generally.

The contribution of word knowledge to the segmentation of connected speech is well documented (e.g., McClelland & Elman, 1986; Norris, 1994). In favorable listening conditions, lexical segmentation cues are prioritized over acoustic, prosodic, and phonotactic cues (Mattys, White, & Melhorn, 2005). Lexical knowledge also facilitates the discovery of novel words. According to the INCDROP model (INCremental Distributional Regularity Optimization; Brent, 1999), the identification of known words in connected speech allows the edges of adjacent (novel) words to be inferred. This strategy has been shown to operate in adults (Dahan & Brent, 1999; White, Melhorn, & Mattys, 2010) and in infants (Bortfeld, Morgan, Golinkoff, & Rathbun, 2005; Sandoval & Gómez, 2016).

In this study, we investigated adults’ ability to use known words to improve the segmentation of new words in a statistical learning (SL) task. In a typical SL experiment, listeners first hear a continuous speech stream made of recurring and coarticulated novel words. A subsequent recognition test assesses how well listeners extract the novel words based on transitional probabilities between syllables (Saffran, Newport, & Aslin, 1996). A vast literature has shown that infants and adults perform above chance on this task (e.g., Romberg & Saffran, 2010).

The presence of known words in a continuous stream can support the extraction of the surrounding novel words, at least in infants (Mersad & Nazzi, 2012). Whether this lexical boost persists in adulthood is unclear. On the one hand, there is evidence that, like infants, adults use existing knowledge to guide learning in SL tasks. For example, Perruchet and Tillmann (2010) demonstrated that the presence of sequences rated as word-like (based on their morphosegmental structure) led to better SL, suggesting that familiarity with sublexical structure may boost segmentation of novel words. Later work by Cunillera and colleagues tested the benefit of quasilexical information by preceding a continuous speech stream with a brief familiarization phase (Cunillera, Càmara, Laine, & Rodriguez-Fornells, 2010; Cunillera, Laine, & Rodriguez-Fornells, 2016). Adults were presented with a 3-min story in which two of the eight (nonsense) words from the artificial language were heard several times in reference to simple items. Familiarization improved subsequent segmentation of the artificial language. Finally, Poulin-Charronnat, Perruchet, Tillmann, and Peereman (2017) found that familiarizing sequences inconsistent with the statistically defined words impaired learning. Thus, short sequences familiarized immediately prior to exposure of an artificial language can constrain segmentation.

On the other hand, it is possible that adults learning unfamiliar languages will not benefit from the presence of real words. Indeed, while recently acquired words can produce lexical boost–like effects in adults (Cunillera et al., 2010, 2016), such items are not equivalent to established, real words. Recently acquired words are not consolidated in the lexicon immediately and they do not activate lexical-semantic networks (e.g., Tamminen & Gaskell, 2008). Activation of these networks by real words can cause a shift in processing mode, with increased reliance on lexical knowledge and reduced attention to bottom-up cues in the speech signal (e.g., Mirman, McClelland, Holt, & Magnuson, 2008). Such a shift in processing mode would be counterproductive in a speech segmentation task, where native language structures are unrelated to the novel input.

Lexical effects are less likely to adversely affect infant learners for several reasons. First, according to the “less is more hypothesis” (Newport, 1988, 1990), limited perceptual and memory functions in children mean that they are better suited to tasks involving signal-driven, componential analysis. Second, since infants have less extensive lexical knowledge than adults, they are less likely to experience shifts in processing mode, and hence, be liable to lexical interference. In fact, it has been argued that a lack of lexical knowledge is precisely what makes children better language learners than adults, with a developing lexicon diverting attention away from low-level regularities (e.g., Birdsong & Molis, 2001; Newport, 1990).

Thus, the goal of this study was to test whether the facilitatory effect of newly familiarized stimuli generalizes to untrained, unprimed, real words. In Experiment 1, listeners heard a 6-min continuous speech stream before completing a recognition task. In one condition, the stream contained four novel words. In another condition, one of the novel words was replaced with a real English word (*tomorrow*). Extraction of the three novel words common to both conditions was compared across conditions. Experiment 2 sought to rule out the possibility that the results of Experiment 1 were due to metrical expectations (number of syllables) rather than lexicality of the known word. To do so, we replaced the trisyllabic known word with a tetrasyllabic known word (*philosophy*). In Experiment 3, we controlled the number of novel words to extract by simply adding a real word to the artificial language rather than substituting a real word for a novel word. To increase sensitivity to differences between conditions, we measured recognition using a 6-point confidence scale between two alternative responses rather than the more conventional forced-choice task.

## Experiment 1

### Method

#### Participants

In all three experiments of this study, participants were native British English young adults from the University of York who received an honorarium or course credit for their participation. The average age of the entire sample was 21 years (range: 18–39). Sixty-three percent were female. All participants had normal or corrected vision and had no known hearing impairment. Ethical approval for all experiments was obtained from the departmental ethics committee. Experiment 1 included 48 participants.

#### Stimuli

##### Familiarization streams

The familiarization phase in the baseline condition consisted of one of two artificial speech streams. Each stream was made of four consonant-vowel (CV)-CV-CV trisyllabic novel words taken from Saffran, Aslin, and Newport (1996; Stream 1: *pabiku*, *tibudo*, *daropi*, *golatu*; Stream 2: *tudaro*, *bikuti*, *budopa*, *pigola*). Although the pool of syllables was common to both streams, the streams differed in how the syllables were assembled into novel words, which was meant to minimize any phonological and phonotactic idiosyncrasies inherent to each stream. Stimulus construction followed Palmer and Mattys (2016). Within each stream, the four novel words were concatenated in a pseudorandom order, ensuring that there were no contiguous novel word repetitions. Each novel word appeared 125 times, for a total of 500 words for each stream. The transitional probability between the syllables within a novel word was 1. It was .33 between syllables spanning word boundaries. The streams were synthesized using an English male diphone database (en1) from the text-to-speech MBROLA software (Dutoit, Pagel, Pierret, Bataille, & van der Vrecken, 1996), with a flat F0 of 120 Hz throughout the stream. Each syllable lasted 240 ms. Stream duration was 6 min.

The streams in the known-word condition were the same as in the baseline condition, except that the syllables of one of the novel words (*tibudo* in Stream 1 and *tudaro* in Stream 2) were replaced with the syllables of the word *tomorrow*. This word was created by MBROLA as a CV-CV-CV string, using the same F0 and duration and coarticulation criteria as those for the novel words.

##### Recognition stimuli

These consisted of pairs of trisyllabic stimuli. In the baseline condition, one stimulus of the pair was one of the four novel words (e.g., *pabiku*). The other stimulus was one of three types of trisyllabic foils. The first type of foil overlapped with the paired novel word by one syllable. These foils were formed by concatenating the third syllable of the novel word with the first two syllables of another novel word (e.g., *kutibu*). The second type of foil overlapped with the paired novel word by two syllables. These foils were formed by concatenating the second and third syllables of the novel word with the first syllable of another novel word (e.g., *bikuti*). We refer to these two types of foil as part-words. They are collapsed in all subsequent analyses because their contrast only serves a counterbalancing function and has no relevant theoretical implication. The third type of foil did not overlap with the paired novel word. It was formed by concatenating syllables from the other three novel words (e.g., *tipila*). Some of these maintained their original position while others did not. We refer to these foils as nonwords. By design, some of the part-word foils in Stream 1 were the novel words of Stream 2, and vice versa. Targets and foils for Experiments 1 and 2 are shown in Table 1.[Table-anchor tbl1]

The test pairs in the known-word condition were those of the baseline condition, except that the syllables making up the novel word *tibudo* in Stream 1 and *tudaro* in Stream 2 were replaced with those making up *tomorrow* in all targets and foils. Note that these substitutions meant that the reciprocal status of the targets and foils across the two streams was partial rather than total. This issue is addressed directly in Experiment 3.

#### Design and procedure

Participants were randomly assigned to the baseline or known-word condition (24 in each). Within each condition, 12 participants heard Stream 1 and 12 heard Stream 2. The familiarization phase was immediately followed by the recognition test. The experiment took place in a sound-attenuated booth. Stimuli were played over Sony MDR V700 headphones at approximately 70 dB SPL. DMDX software (Forster & Forster, 2003) was used for presenting stimuli and recording responses.

Before the familiarization phase of both the baseline and known-word conditions, participants were told that they would hear an artificial language played over the headphones, and that they should try to discover what the words of the language are.

In the recognition test, each target was paired with each of the three foil types, yielding 12 pairs. Each pair was played in both a target-foil and foil-target order. Thus, the test phase comprised 24 trials. Pair order was randomized across participants. The two members of a pair were separated by a 500-ms silent interval. Participants were asked to rate their confidence in which of the two stimuli was a word of the language using one of three levels of confidence for each alternative (highly confident [3], moderately [2], and somewhat [1]). The alternatives were presented visually as 3 2 1–1 2 3 on a computer monitor, with the numbers on the left corresponding to the first stimulus of the pair and those on the right to the second stimulus. The next pair was presented 1 s following a response, or after a 10-s deadline.

To ensure that the CV-CV-CV string *tomorrow* created by MBROLA was perceived as the intended word, participants in the known-word condition were asked to transcribe this sequence at the end of the experiment.

### Results

**Figure 1 fig1:**
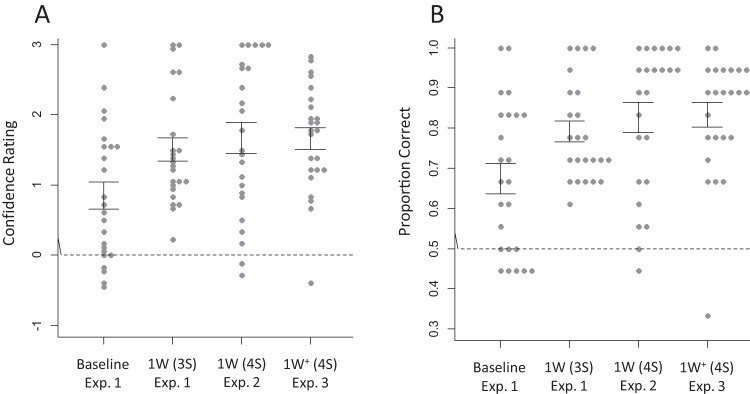
(A) Confidence ratings (negative values = incorrect; positive values = correct) in Experiments 1, 2, and 3. Ratings are averaged across streams and trial types, and across the three common novel words within each stream. They exclude known words (*tomorrow* and *philosophy*) or substituted novel words (*tibudo* and *tudaro*). Gray dots represent scores of individual participants. Error bars represent the standard errors of the means. The dotted line represents chance level. (B) Same as in panel A, with ratings converted to binary data (−3, −2, −1 = 0 [incorrect]; 1, 2, 3 = 1 [correct]). Exp. = experiment; 1W (3S) = trisyllabic known word (*tomorrow*) replacing one of the four novel words; 1W (4S) = tetrasyllabic known word (*philosophy*) replacing one of the four novel words; 1W^+^ (4S) = tetrasyllabic known word (*philosophy*) added to the four novel words.

The transcription task confirmed that all participants in the known-word condition correctly identified the word *tomorrow*. Correct responses were scored as positive (1, 2, 3) and incorrect responses as negative (−1, −2, −3). For comparison with conventional measures of statistical learning, accuracy was also calculated on converted binary data. Here, each positive rating (1, 2, 3) was converted into a correct score (1) and each negative rating (−1, −2, −3) was converted into an incorrect score (0). The by-participant ratings are plotted in Figure 1A and the converted binary scores in Figure 1B.[Fig-anchor fig1]

In order to produce a meaningful comparison between the baseline and known-word conditions, analyses (ratings and binary scores) were performed on the recognition accuracy of only the novel words that were common to the two conditions (Stream 1: *pabiku*, *daropi*, *golatu*; Stream 2: *bikuti*, *budopa*, *pigola*). Thus, in the baseline condition, we did not consider trials including *tibudo* (Stream 1) or *tudaro* (Stream 2), as these were replaced with *tomorrow* in the known-word condition. Likewise, in the known-word condition, we did not include trials testing the recognition of *tomorrow*. Performance on *tibudo*/*tudaro* and *tomorrow* is reported separately.

The rating data were analyzed using generalized mixed-effect models. The converted binary scores were analyzed using logistic mixed-effect models. The fixed factors were Familiarity (baseline vs. known-word), Stream (Stream 1 vs. Stream 2), and Foil Type (part-words vs. nonwords). The two levels of each fixed factor were coded as −1 and +1. Main effects and interactions were assessed by comparing a full model with a model lacking the critical effect or interaction using the likelihood ratio test. For all models, we included by-subject and by-item random intercepts. Random slopes were not entered because these prevented the models from converging. The outcome of the statistical analyses is shown in Table 2.[Table-anchor tbl2]

Ratings and binary displayed very similar patterns. Performance was better in the known-word than baseline conditions, which shows that introducing a known word improved extraction of the novel words. The Stream effect indicates that Stream 1 was easier than Stream 2, which might reflect a slight imbalance in familiarity between the syllable sequences of the two streams, a frequent occurrence with artificial languages. The Foil Type effect shows that the novel words were easier to discriminate from the nonword foils than from the part-word foils, which confirms listeners’ expected sensitivity to different degrees of transitional probability. Interaction terms did not improve model fit. Finally, performance was higher for the known word (*tomorrow*) than for the novel words it replaced (*tibudo* or *tudaro*), in terms of ratings, 2.27 versus .26, *b* = 2.013, *SE* = .341, χ^2^(1) = 9.55, *p* = .002, and binary scores, .87 versus .57, *b* = 2.622, *SE* = .704, χ^2^(1) = 9.11, *p* = .002, which confirms that the known word was effectively recognized.

Finally, an analysis of confidence ratings restricted to the correct responses showed no difference between the baseline and known-word conditions, 2.26 versus 2.40, respectively, *b* = .141, *SE* = .125, χ^2^(1) = 1.33, *p* = .25. However, participants’ average confidence in correct responses was correlated with their average accuracy (binary data) in the known-word condition (*r* = .46, *p* = .02), but not in the baseline condition (*r* = .31, *p* = .14). Thus, although the presence of a known word did not improve confidence, it might have increased awareness of the learning process in those participants who took most advantage of the known-word boost.

### Discussion

Experiment 1 shows that substituting one of the novel words of the artificial language with a known word significantly improves extraction of the other novel words. This result is consistent with the idea that known words provide boundary cues for surrounding novel words. It does not support the hypothesis that known words draw attention away from the computation of Transitional Probabilities (TPs).

However, it is possible that the improvement was driven by the metrical expectation induced by the known word. Recognizing *tomorrow* could have led listeners to accurately assume that the other words of the stream were trisyllabic as well. Indeed, Hoch, Tyler, and Tillmann (2013) have found that using words of the same length in artificial languages improves learning. Therefore, in Experiment 2, we replaced the trisyllabic word *tomorrow* with the tetrasyllabic word *philosophy*. If the lexical boost was the result of metrical expectations, using a tetrasyllabic word should attenuate or eliminate the effect found in Experiment 1. However, if a known word promotes segmentation by cueing the boundaries of the adjacent novel words, Experiment 2 should replicate Experiment 1.

## Experiment 2

### Method

This experiment included 24 participants. The streams and test pairs were identical to those in the known-word condition of Experiment 1, except that *tomorrow* was replaced with *philosophy*. Like *tomorrow*, *philosophy* was generated with MBROLA and coarticulated with the rest of the stream. Because *philosophy* was four syllables long (i.e., 960 ms instead of 720 ms), the total duration of the streams was 6 min 30 s.

The structure of the test phase and the number of pairs were the same as in Experiment 1, but the inclusion of a tetrasyllabic word led to some changes. As shown in Table 1, some of the foils contained syllables from *philosophy*. The more substantial change was that *philosophy* was paired with tetrasyllabic foils to prevent responses from being influenced by a length bias. Part-word foils paired with *philosophy* started with the last two or three syllables of *philosophy* and ended with the first two or first syllable(s) of another stimulus (e.g., *sophypabi*, *losophypa*). Nonword foils paired with *philosophy* were made of a combination of four syllables drawn from the three novel words (two syllables from one of the novel words and one syllable from each of the other two) that never appeared contiguously in the stream. Note that performance on pairs containing *philosophy* is of secondary importance, since those pairs were not included in the main analyses. The rest of the design and procedure was the same as in Experiment 1.

### Results

The transcription task confirmed that all participants correctly identified the word *philosophy*. As shown in Figure 1A-B and Table 2, a comparison between the known-word condition of Experiment 2 and the baseline condition of Experiment 1 showed a Familiarity effect, replicating the lexical boost in Experiment 1. As before, the Stream effect indicated slightly better learnability of Stream 1. The Foil Type effect confirmed that the novel words were easier to discriminate from nonword than part-word foils.

None of the interaction terms were significant, except for Stream × Foil Type, which showed an attenuation of the Stream effect in the nonword compared to the part-word foils. As in Experiment 1, ratings were higher for the known word (*philosophy*; 2.26) than for the novel words it replaced (*tibudo* or *tudaro*; 0.26) in terms of ratings, *b* = 2.006, *SE* = .398, χ^2^(1) = 9.04, *p* = .003, and binary scores (.90 vs. .57), *b* = 2.956, *SE* = .752, χ^2^(1) = 9.64, *p* = .002.

There was no difference between the two known-word conditions (Experiment 1 *tomorrow* vs. Experiment 2 *philosophy*) on either ratings, *b* = .170, *SE* = .265, χ^2^(1) = .44, *p* = .51, or binary scores, *b* = .408, *SE* = .354, χ^2^(1) = 1.37, *p* = .24. Performance on the trials containing the real word was equally high for *tomorrow* and *philosophy*, on either ratings, *b* = .012, *SE* = .620, χ^2^(1) = .00, *p* = .98, or binary scores, *b* = .295, *SE* = 1.582, χ^2^(1) = .03, *p* = .85.

As before, confidence in correct responses was comparable in the baseline and know-word conditions (2.26 vs. 2.47, respectively), *b* = .188, *SE* = .128, χ^2^(1) = 2.23, *p* = .13, but a significant correlation between confidence and accuracy in the known-word condition only (*r* = .56, *p* = .004) suggested increased awareness of the learning process in that condition.

### Discussion

Experiment 2 confirms the lexical boost in Experiment 1, independent of length expectations that the known word might induce. However, the results might have been influenced by two design features. First, while replacing one of the novel words with a known word ensured comparable cross-boundary syllable transition probabilities, it could have reduced the memory load involved in storing the novel words (from four to three). Second, by design, the introduction of a known word affected the construction of the foils in the test phase, making the test phases in the baseline and known-word conditions different and hence complicating the interpretation of the comparison.

In Experiment 3, we addressed these caveats by adding a word (*philosophy*) to the four novel words of the baseline stream rather than replacing one of them. Although this design led to a slight change in cross-boundary transitional probabilities, it kept the number of novel words in the baseline and known-word conditions the same (four) and enabled us to use the same test pairs in both conditions.

## Experiment 3

### Method

This experiment included 24 participants. The speech streams were those used in the baseline condition of Experiment 1, except that the word *philosophy* was added to the streams. Like each novel word, *philosophy* was heard 125 times, interspersed in random positions in the stream. The total duration of each stream was 8 min. The test phase was that of the baseline condition of Experiment 1. The word *philosophy*, or portions of that word, were never heard in any of the test stimuli. The rest of the design and procedure was the same as in Experiment 1.

### Results and Discussion

**Figure 2 fig2:**
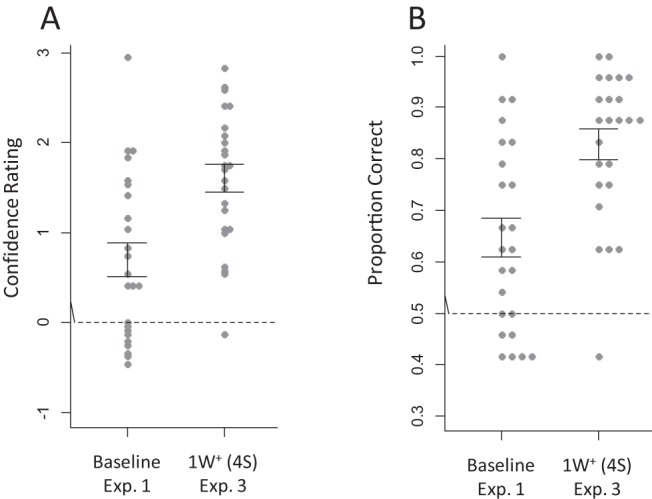
Results of Experiment 1 (baseline condition) and Experiment 3 averaged across all four novel words within each stream. Gray dots represent scores of individual participants. Error bars represent the standard errors of the means. The dotted line represents chance level. (A) Confidence ratings (negative values = incorrect; positive values = correct). (B) Ratings converted to binary data (−3, −2, −1 = 0 [incorrect]; 1, 2, 3 = 1 [correct]). Exp. = experiment; 1W^+^ (4S) = tetrasyllabic known word (*philosophy*) added to the four novel words.

The transcription task confirmed that all participants correctly identified the word *philosophy*. Figure 2A-B shows the ratings and converted binary scores averaged across all four novel words in Experiment 3 and in the baseline condition of Experiment 1. Main statistical analyses are reported in Table 2. Performance on both ratings and binary data was higher in the known-word than baseline condition, showing once again better extraction of novel words in the presence of a known word. There was no stream effect this time, but a Foil Type effect revealed higher ratings against nonword than part-word foils. There was no interaction between Familiarity and Stream, but a marginal interaction between Familiarity and Foil Type in the rating data indicated a slightly stronger Familiarity effect against the part-word foils than against the nonword foils. Participants’ confidence in their correct responses was not affected by the presence of the known word (2.19 vs. 2.29, respectively), *b* = .115, *SE* = .102, χ^2^(1) = 1.27, *p* = .26. As before, however, confidence and accuracy correlated in the known-word condition (*r* = .65, *p* = .001), but not in the baseline condition (*r* = .33, *p* = .12).[Fig-anchor fig2]

For comparability across all three experiments, the results of Experiment 3, restricted to the common novel words in Experiments 1–2, are plotted in Figure 1. In clearly replicating the lexical benefit found in Experiments 1–2, these results rule out the possibility that this effect was due to a reduced memory load (fewer novel words to learn) or differences in design features between test phases.

## General Discussion

In three experiments, we investigated whether known words can boost the segmentation of continuous artificial speech. Experiment 1 demonstrated that replacing a novel word with a real word improved performance in a subsequent recognition memory test. Experiment 2 showed that this improvement was not due to the known word providing a metrical chunking cue which would have allowed listeners to infer the length of the novel words. Experiment 3 established that performance was improved even when a familiar word was added to the stream rather than replaced one of the novel words, suggesting that the lexical boost in Experiments 1–2 was not due to the smaller number of novel words to extract compared to the baseline condition. Although performance was higher for trials involving word/nonword than word/part-word test pairs, the lexical boost was broadly comparable in both cases, indicating that it generalized across trial types. Finally, an analysis of confidence ratings showed that, although participants in the baseline condition were as confident in their responses as those in the known-word conditions, confidence only correlated with accuracy in the known-word conditions. Therefore, the presence of a known word in the stream not only increased accuracy, but also made participants more aware of their performance. A question for future research is whether this pattern would hold if learning was incidental (passive listening to the stream) rather than intentional.

Overall, these findings demonstrate for established real words the segmentation boost previously found for word-like sequences (Perruchet & Tillmann, 2010) and familiarized novel words (Cunillera et al., 2010, 2016; Poulin-Charronnat et al., 2017). There was no evidence that known words caused a shift in processing mode, or otherwise disrupted performance in adult learners. The fact that the effect was independent of the length of the known word is critical not only because it shows that lexical knowledge outweighs any perceived rhythmic grouping of the stream, not assessed in Cunillera et al.’s studies, but also because mixed-length words are the norm, rather than an exception, in natural language. In future research, it will be necessary to pit the lexical boost against explicit rhythmic cues (e.g., pitch, lengthening) to test the robustness of lexically driven segmentation relative to sublexical cues. Data from infant (Sandoval & Gómez, 2016) and adult segmentation research (e.g., Mattys et al., 2005) suggest that known words should override any conflicting rhythmic cues.

The benefit of known words for segmentation can be accounted for by several mechanisms. In the INCDROP model (Brent, 1999), it can be interpreted entirely in terms of lexically driven segmentation. Unknown portions of speech between known words are initially encoded as single units and these are subsequently divided into smaller units when interrupted by known words later in the stream. This incremental-subtraction process does not in principle require the computation of TPs. Therefore, for INCDROP, known words would not so much enhance reliance on TPs as they would make their computation unnecessary.

Another model, PARSER (Perruchet & Vinter, 1998) also provides an account that does not require explicit computation of TPs, but it overcomes issues of cognitive plausibility (e.g., working memory constraints) by assuming that the initial chunks are of cognitively tractable length rather than portions between known words. Frequent chunks are reinforced and these are able to drive segmentation by reducing the activity of less frequent chunks. PARSER predicts that known words should boost segmentation in the same way that highly activated chunks do, thereby shortening (or at least optimizing) the initial chunking stage.

An alternative to both INCDROP and PARSER is an account of lexically driven segmentation that coexists with, rather than eschews, the computation of TPs. Here, the identification of a familiar word would allow the initial segments of the following word (and possibly the final segments of the preceding word) to be firmly identified. These flagged segments, in turn, would more effectively highlight the boundaries of novel words when encountered subsequently. Such a flagging mechanism is consistent with the privileged status of word initial segments sometimes assumed in spoken-word recognition models (e.g., Content, Kearns, & Frauenfelder, 2001; Marslen-Wilson & Zwitserlood, 1989).

Finally, the above alternatives contrast with a purely attentional account. Here, known words would generate global attention to and engagement with the stream as a whole. Attention has been shown to significantly affect TP computation (e.g., Fernandes, Kolinsky, & Ventura, 2010; Toro, Sinnett, & Soto-Faraco, 2005). This attention-based explanation is a challenge to the claim that known words highlight the offset and onset of adjacent novel words, as it predicts no marked difference for novel words that are contiguous versus noncontiguous with the known words.

In summary, the findings presented here provide clear evidence that familiar-word identification promotes the discovery of novel words in connected speech. Future research will focus on gaining a better understanding of the way in which familiar words interact with bottom-up computation. Of particular relevance is whether lexically driven segmentation operates independently of TPs, enhances sensitivity to TPs, or draws resources away from them. Likewise, it remains to be established whether known words simply reduce task demand or whether they contribute to segmentation by providing a distinct processing contribution, such as flagging likely word onsets.

## Figures and Tables

**Table 1 tbl1:** Targets and Paired Foils in the Test Phase of Experiments 1–2

			Experiment 1		Experiment 2
Stream	Target	Foil	Baseline	1 word (TOMORROW)	1 word (PHILOSOPHY)
1	tibudo	PW1	dopabi	ROpabi	SOPHYpabi
	(or TOMORROW)	PW2	budopa	MOROpa	LOSOPHYpa
	(or PHILOSOPHY)	NW	rotula	rotula	rotulabi
	pabiku	PW1	kutibu	kuTOMO	kuPHILO
		PW2	bikuti	bikuTO	bikuPHI
		NW	tipila	TOpila	PHIpila
	daropi	PW1	pigola	pigola	pigola
		PW2	ropigo	ropigo	ropigo
		NW	kudobi	kuRObi	kuPHYbi
	golatu	PW1	tudaro	tudaro	tudaro
		PW2	latuda	latuda	latuda
		NW	bupada	MOpada	LOpada
2	tudaro	PW1	ropigo	ROpigo	SOPHYpigo
	(or TOMORROW)	PW2	daropi	MOROpi	LOSOPHYpi
	(or PHILOSOPHY)	NW	tidogo	tigodo	tidogopa
	bikuti	PW1	tibudo	tibudo	tibudo
		PW2	kutibu	kutibu	kutibu
		NW	bulapa	bulapa	bulapa
	budopa	PW1	pabiku	pabiku	pabiku
		PW2	dopabi	dopabi	dopabi
		NW	birotu	biROTO	biPHYPHI
	pigola	PW1	latuda	laTOMO	laPHILO
		PW2	golatu	golaTO	golaPHI
		NW	dakudo	MOkudo	LOkudo
*Note.* Portions of the known words making up the foils are shown in capital letters. PW1 = partword foil with a one-syllable overlap with the paired target (two-syllable overlap in Experiment 2); PW2 = partword foil with a two-syllable overlap with the paired target (three-syllable overlap in Experiment 2); NW = nonword foil with no syllable overlap with the target.					

**Table 2 tbl2:** Summary of Main Statistical Analyses for Experiments 1–3

Effect	*b*	*SE b*	χ^2^(1)	*p*
Experiment 1 (baseline vs. tomorrow)				
Ratings				
Familiarity	.654	.230	7.32	.007
Stream	−.641	.247	6.50	.01
Foil type	−.712	.134	27.75	.000
Familiarity × Stream	.212	.484	.21	.65
Familiarity × Foil Type	−.036	.268	.02	.89
Stream × Foil Type	−.299	.268	1.25	.26
Familiarity × Stream × Foil Type	.100	.537	.03	.85
Binary scores				
Familiarity	.675	.257	6.38	.01
Stream	−.685	.269	5.61	.02
Foil type	−1.001	.192	30.02	.000
Familiarity × Stream	−.006	.514	.00	.99
Familiarity × Foil Type	−.429	.399	1.16	.28
Stream × Foil Type	−.214	.390	.30	.59
Familiarity × Stream × Foil Type	.479	.815	.34	.56
Experiment 2 (baseline vs. philosophy)				
Ratings				
Familiarity	.823	.286	8.36	.004
Stream	−.728	.332	4.77	.03
Foil type	−.622	.128	23.42	.000
Familiarity × Stream	.040	.566	.00	.94
Familiarity × Foil Type	.140	.254	.31	.58
Stream × Foil Type	−.708	.254	7.72	.005
Familiarity × Stream × Foil Type	−.712	.509	1.97	.16
Binary scores				
Familiarity	1.131	.359	9.41	.002
Stream	−.895	.442	3.66	.06
Foil type	−.919	.200	22.29	.000
Familiarity × Stream	−.291	.714	.17	.68
Familiarity × Foil Type	−.017	.413	.00	.96
Stream × Foil Type	−.851	.401	4.33	.04
Familiarity × Stream × Foil Type	−1.219	.833	2.07	.15
Experiment 3 (baseline vs. philosophy)				
Ratings				
Familiarity	.902	.241	12.71	.000
Stream	−.314	.348	.90	.34
Foil type	−.535	.108	24.23	.000
Familiarity × Stream	.475	.483	1.02	.31
Familiarity × Foil Type	.414	.216	3.69	.05
Stream × Foil Type	−.409	.216	3.58	.06
Familiarity × Stream × Foil Type	.652	.431	2.28	.13
Binary scores				
Familiarity	1.130	.300	12.61	.000
Stream	−.372	.379	.94	.33
Foil type	−.789	.166	23.57	.000
Familiarity × Stream	.298	.596	.25	.62
Familiarity × Foil Type	.296	.339	.73	.39
Stream × Foil Type	−.640	.331	3.67	.06
Familiarity × Stream × Foil Type	.324	.676	.22	.64
